# Recent Advances in Comprehending the Signaling Pathways Involved in the Progression of Breast Cancer

**DOI:** 10.3390/ijms18112321

**Published:** 2017-11-03

**Authors:** Andrea Nicolini, Paola Ferrari, Lucrezia Diodati, Angelo Carpi

**Affiliations:** 1 Department of Oncology, Transplantations and New Technologies in Medicine, University of Pisa, 56126 Pisa, Italy; paolaferrari2266@libero.it (P.F.); lucreziadio@live.it (L.D.); 2 Department of Clinical and Experimental Medicine, University of Pisa, 56126 Pisa, Italy; angelo.carpi@med.unipi.it

**Keywords:** breast cancer, cancer progression, signaling pathways, personalized medicine, calcium sensing receptor, caveolae, hypoxia inducing factors

## Abstract

This review describes recent advances in the comprehension of signaling pathways involved in breast cancer progression. Calcium sensing receptor (CaSR), caveolae signaling, signaling referred to hypoxia-inducing factors and disturbances in the apoptotic machinery are related to more general biological mechanisms and are considered first. The others refer to signaling pathways of more specific biological mechanisms, namely the heparin/heparin-sulfate interactome, over-expression of miRNA-378a-5p, restriction of luminal and basal epithelial cells, fatty-acid synthesis, molecular pathways related to epithelial to mesenchimal transition (EMT), HER-2/neu gene amplification and protein expression, and the expression of other members of the epithelial growth factor receptor family. This progress in basic research is fundamental to foster the ongoing efforts that use the new genotyping technologies, and aim at defining new prognostic and predictive biomarkers for a better personalized management of breast cancer disease.

## 1. Introduction

It is largely known that cancer is a complex disease sustained by many genetic and epigenetic alterations. These alterations, at least in part, differ in different types, within the same type of cancer and even within each cancer. This main feature, termed “spatial heterogeneity” [1,2], occurs in addition to “temporal heterogeneity” [3,4]. In fact, concomitant with tumor growth, genetic and epigenetic alterations are occasionally acquired or are induced by the microenvironmental signaling or are due to therapeutic pressure. On the other hand, the spatial and temporal heterogeneities entail changes in the local microenvironment [5,6] that also likely affect the signaling pathways related to breast cancer progression. This makes cancer an evolving disease hard to be understood and even more to be cured in the advanced stages. Genetic alterations and the contextual signaling by the microenvironment give rise to an intricate network of pathological mechanisms and molecular pathways. In breast cancer, so far many have been elucidated while many others are either under investigation or to be investigated [7]. Here, novel findings that can help in a better comprehension of some widespread and others inherent to more specific biological mechanisms involved in breast cancer progression are reported. Most described signaling pathways occur in normal and breast cancer cells including Luminal A, Luminal B, triple negative, and HER2 positive molecular subtypes, according to the current classification of breast cancer.

## 2. Alterations of Signaling Pathways in Some Widespread Biological Mechanisms Involved in Breast Cancer Progression

### 2.1. The Calcium-Sensing Receptor (CaSR) Signaling

#### 2.1.1. Normal Parathyroid and Mammary Glands

The calcium ion (Ca^2+^) plays a key role in maintaining calcium homeostasis, and Ca^2+^ is the main physiological ligand of G protein-coupled receptor (GPCR). This receptor, also known as extracellular calcium-sensing receptor (CaSR), takes part of class C of the GPCR superfamily [8]. It is mostly expressed in parathyroid glands, where it regulates the parathyroid hormone (PTH) secretion in response to changes in extracellular free calcium [9]. In these glands, high Ca^2+^ levels activate CaSR and reduce the release of the calcium-retaining hormone, PTH, and vice versa occurs in the case of hypocalcemia. In this last instance, following CaSR inactivation, PTH is released and this accounts for an increase of Ca^2+^ recovery both at a renal and intestinal level in addition to mobilization of Ca^2+^ from the bony skeleton. This mechanism allows Ca^2+^ to be maintained within the physiological range of 1.1–1.3 mM [10].

In the parathyroid glands and other tissues, PTH inhibition due to CaSR activation occurs through trimeric G protein G αq/11 signaling. In mice without G αq/11 signaling, high PTH levels occur, and genetic mutations of the CaSR demonstrate its relevance in maintaining Ca^2+^ homeostasis [11]. CaSR is expressed in a few other cell types, such as astrocytes, hepatocytes, cytotrophoblasts, blood vessels, and in human breast epithelial ductal cells which are not involved in Ca^2+^ homeostasis [12] Interestingly, in these sites, CaSR often governs the secretion of parathyroid hormone-related protein (PTHRP) [13], which is a growth factor using the same Type 1 PTH/PTHRP receptor (PTH1R). In a normal breast, CaSR expression was first documented by Cheng and coworkers [14], and successive findings in mice confirmed the observation [15]. Thereafter, some studies ruled out a relevant CaSR role in governing morphological breast development or differentiation, while others showed that it affected calcium transportation, PTHRP production and systemic calcium metabolism during lactation [12]. In normal cells of the mammary gland, CaSR binds to Gαi to decrease cAMP production that in turn inhibits Pthlh gene expression and PTHRP secretion [16] In addition, it was found that in most differentiated cell types an activated CaSR induced PTHRP production, while it was suppressed in normal mammary gland. CaSR loss in normal mammary epithelial cell provoked an increased Pthlh mRNA expression, high milk PTHRP levels, and an augmented PTHRP secretion into the maternal circulation. This supports the notion of a negative feedback between calcium delivery to the mammary gland and PTHRP production by epithelial breast cells during breastfeeding. Conversely, CaSR seems to govern the transport of calcium from circulation into milk by a positive feedback loop affecting the activity of the plasma membrane calcium-ATPase 2 (PMCA2) pump. This pump is expressed on the surface of mammary epithelial cells by which it carries 60–70% of milk calcium into the acinar lumen [17]. On the other hand, circulating PTHRP acts on osteoblasts to increase the number and activity of osteoclasts through the receptor activator of nuclear factor-κB ligand (RANK/RANKL) pathway. When calcium is easily available in the diet, the activated CaSR increases the PMCA2 pump activity with more calcium transported into milk but suppresses PTHRP release into milk and the systemic circulation. Therefore, less calcium resorption from skeletal stores occurs. When dietary calcium is less abundant, the calcium transport by PMCA2 pump activity is slowed, and more PTHRP is produced. This increases the bone resorption with more calcium available from skeletal stores.

#### 2.1.2. Breast Cancer Cells

##### CaSR Expression and the Effects of CaSR Signaling on Cell Proliferation and Death

In the above-mentioned report from Cheng and coworkers [14] and in other studies, CaSR was found to be expressed both in human breast cancers and in MCF-7 and MB-231 breast cancer cell lines [18]. However, in some studies, CaSR was overexpressed in breast tumors as compared to normal mammary cells [19], while, in others, lower levels were found expressed in human breast cancers than in normal breast tissue [20,21]. Breast cancer cell lines had over-expressed CaSR compared to nonmalignant breast cell lines [22,23]. A high expression of CaSR levels was also found to be associated with bone metastases [24], directly correlated with N positive, and inversely with Pr positive status [25], and CaSR SNP (rs112594756) was reported to be associated with ER-negative status for case-only analyses [26]. These controversial findings cannot be easily explained because the mechanisms regulating the CaSR expression in breast cancer cells are not well understood, although in an experimental study conducted in MCF-7 and MDA-MB-231 breast cancer lines, CaSR was found to be positively regulated by BRCA1 [27]. Controversial findings have also been reported regarding the effects of CaSR signaling on proliferation and cell death. In one study, a pathway activated by CaSR and involving membrane metalloproteinases (MMPs), epidermal growth factor receptor (EGFR) stimulation, ERK1/2 phosphorylation, up-regulation of the transient receptor potential channel 1 (TRPC1) promoted cell proliferation [28], and further data supported the same signaling mechanism [29,30]. In another study carried out in MCF-7 and MDA-MB-231 breast cancer lines, CaSR activation was hypothesized to stimulate cell proliferation by the increase of the phosphocholine production and the expression of choline kinase through activation of Gα12 and Rho [22]. In addition, in this instance, other findings supported the same hypothesis [31,32]. Two different experimental studies, the former in MCF-7 and MDA-MB-231, and the latter in ER-positive MCF-7 and ER-negative MDA-MB-435 human breast cancer cells, showed that varying the extracellular concentrations of Ca^2+^ between 0.5 and 10 mM no [18] or decreased [33] cell proliferation occurred. Various Ca^2+^ concentrations, used in the different experiments or the existence of complex and context-dependent downstream signaling, have been supposed to interpret these contradictory findings. It was also seen that unlike in normal breast cells, where activated CaSR couples to Gαi and inhibit adenylyl cyclase with the cAMP decrease, in breast cancer cells CaSR couples to Gαs and stimulate cAMP production. The molecular mechanisms of the G-protein switching in response to the malignant transformation are unclear. Nevertheless, as *Pthlh* gene is regulated by a cAMP-response element, it derives that the different CaSR Gα preference following CaSR activation accounts for a decreased and an increased PTHRP production in normal and malignant breast cancer cells respectively. Consistently, a positive correlation in rats and human breast cancers between Casr and Pthlh at mRNA and protein level has been found, while after disrupting the *Casr* gene in mammary tumors in mice, PTHRP mRNA levels also significantly decreased. Moreover, in BT474 and MDA-MB-231 cells, CaSR or PTHRP knocking down in the presence of elevated concentrations of calcium levels induced cell death and inhibited proliferation.

##### CaSR-Nuclear PTHRP Pathway

**Figure 1:**
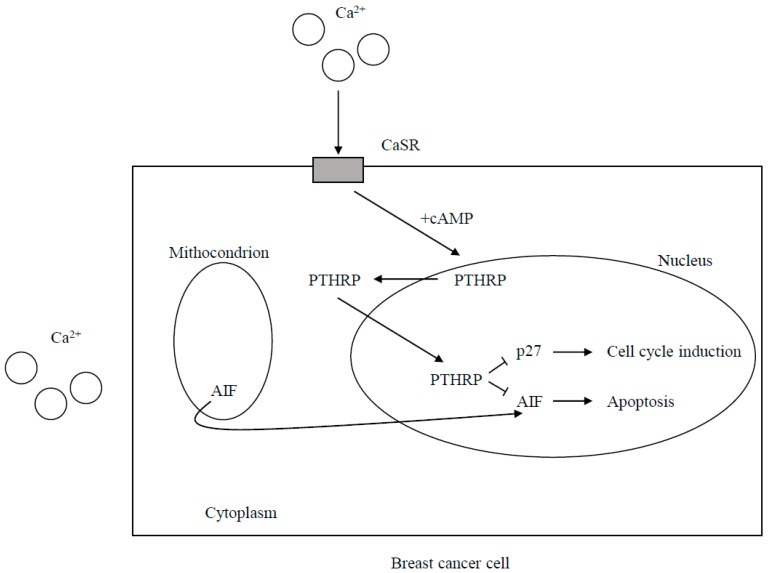
Calcium sensing receptor (CaSR)-nuclear PTHRP pathway. CaSR activation through cAMP levels increases PTHRP production. The increased PTHRP at nuclear level decreases the expression of cell cycle inhibitor p27^kip1^ and inhibits accumulation of apoptosis activator AIF (apoptosis inducing factor). Therefore, proliferation and cell survival are promoted (also see text).

**Figure 2:**
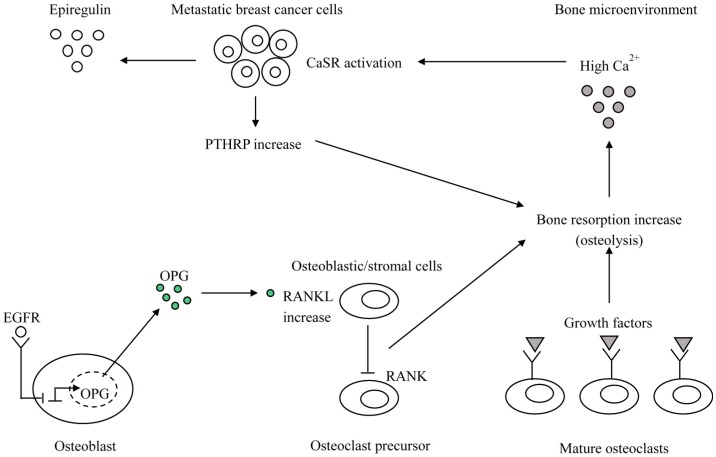
The vicious cycle of bone metastasis and the CaSR-epiregulin axis. At the metastatic bone microenvironment, the elevated Ca^2+^ levels activated CaSR in metastatic breast cancer cells stimulate PTHRP secretion that stimulates osteoblasts (Ob) to produce more RANKL thus driving more osteolysis and release of growth factors (GF) from the bone matrix. As a result, a feed forward vicious cycle of bone resorption tumor growth and osteolysis establishes. In addition, CaSR activation induces epiregulin synthesis and secretion. Epiregulin through its receptors expressed by osteoblasts inhibits OPG synthesis, thus favoring interactions between RANKL expressed by osteoblasts and the receptor RANK expressed by immature osteoclasts. This again feeds the vicious cycle (also see text).

A CaSR-nuclear PTHRP pathway has been hypothesized to account for these findings. In fact, the effects of the CaSR activation on tumor growth should be due to the increased PTHRP value that at nuclear level reduces the expression of the cell cycle inhibitor p27kip1 and prevents nuclear accumulation of apoptosis-inducing factor (AIF), which promotes apoptosis [12] (Figure 1). The above-mentioned CaSR over-expression was shown to be associated with bone metastases. Moreover, it promotes osteolytic secondary bone involvement. At the metastatic site, an increased bone resorption by cancer cells releases a great amount of Ca^2+^ into the local microenvironment. The increased PTHRP secretion in response to the high Ca^2+^ concentrations stimulates in a paracrine way osteoblasts to produce more RANKL, therefore, inducing more osteolysis and releasing growth factors (TGF-β, IGFs, and FGFs) from the bone matrix. They stimulate tumor cell growth and/or increased PTHRP secretion, provoking a vicious cycle of osteolysis. However, the intracrine CaSR-nuclear PTHRP pathway that directly stimulates tumor cell proliferation and allows the cells to survive in spite of the elevated extracellular calcium concentrations is also promoted. Moreover, activated CaSR increases the activity of the PMCA2 calcium pump to protect the breast cancer cells from calcium-mediated apoptosis and governs the TRPC1 calcium channel which can stimulate proliferation. It is currently known that the existence of this CaSR-PTHRP axis could provide further opportunities for novel treatments of breast cancer metastases. In a recent experimental study [34] on MDA-MB-231 cells, estrogen-independent breast cancer cell line prone to induce osteolytic metastasis were transfected with plasmids containing full-length wild-type CaSR. Then these transfected cells were intratibially injected into Balb/c-nude mice. It was found that CaSR overexpression significantly increased the osteolytic potential of MDA-MB-231 cells, likely through epiregulin-mediated osteoprotegerin downregulation. In fact, the overexpressed CaSR increased synthesis and secretion of epiregulin which in turn inhibited the osteoprotegerin synthesis through fixation on its receptor. The diminished osteoprotegerin in bone microenvironment favored the interaction between RankL expressed in osteoblasts and its receptor RANK expressed by immature osteoclasts. This promoted osteoclastogenesis and increased the rate of the bone turnover, thus feeding the vicious circle (Figure 2). However, authors observed that neutralizing antibody targeting epiregulin did not entirely block CaSR mediated decrease in osteoprotegerin. Thus, they also suggested a transactivation of EGFR by the activated CaSR leading to ERK phosphorilation and PTHRP stimulation. The EGFR transactivation and the following increased PTHRP secretion could indirectly worsen osteoprotegerin inhibition in osteoblasts. The authors support this hypothesis with the findings by Zhu and coworkers [35] reporting that EGF-like ligands induce osteoclast production in co-cultures of osteoblastic cells and bone marrow macrophages through the down-regulation of osteoblast osteoprotegerin and monocyte chemotactic protein 1(MCP-1).

### 2.2. The Caveolae and Signaling

#### 2.2.1. Normal Cells

Caveolae are 50–100 nm invaginations of the plasma membrane and the most investigated subset of lipid rafts. Lipid rafts can be planar (flat) or non-planar (invaginated also known as caveolae). Caveolin and cavin proteins are necessary to form the caveolae, while other proteins have scaffolding functions in planar lipid rafts. Many signaling proteins need a covalent lipid change for targeting to lipid rafts; caveolin 1 (CAV1) and CD36 (platelet glycoprotein 4) are palmitoylated membrane proteins targeted to lipid rafts [36,37]. Caveolar lipid rafts act as signaling organelles and account for plasma membrane heterogeneity in normal and tumor cells. They are involved in signal transduction, cholesterol transport and endocytotic internalization of proteins. Caveolins expression ranges in the different cell types but the total lack of caveolins is rare; as key regulators of signal transduction, caveolins have a scaffolding domain for binding to signaling proteins the function of which they modulate. For example, Cav1 inhibits basal activation of Src and HRAS. The caveolin gene family includes *CAV1*, *CAV2*, and *CAV3* with *CAV1* and *CAV2* sharing a wide expression distribution, while *CAV3* is specific to muscle. The cavin family, another main caveolar constituent, includes four members that aggregate to form oligomeric complexes. Cavin 1 (a polymerase I and transcript release factor (PTFR)) is necessary for caveolae formation. Caveolae are also configured as rosettes or caveolar clusters which provide a heterogeneities [38]. Compartmentalization of signal transduction proteins and account for membrane and signal transduction Environmental factors (hyperosmotic stress, metaphase or disruption of integrin-mediated attachment to the extracellular matrix) can regulate the expression of caveolae, and cholesterol is fundamental for their formation. Cholesterol governs *CAV1* transcription, and caveolins are cholesterol-binding proteins that carry lipid rafts between the Golgi, the plasma membrane, and the intracellular organelles [39]. Therefore, caveolae are likely lipid raft stores governing the assembly of functional signaling complexes [40]. Caveolae and *CAV1* deficiencies are associated with decreased cholesterol transportation [40], its accumulation in mitochondrial membranes, mitochondrial dysfunction, and aerobic glycolysis [41].

#### 2.2.2. Breast Cancer Cells

**Figure 3:**
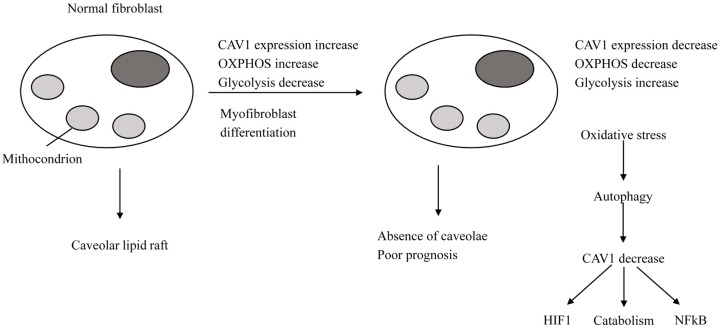
*CAV1* expression in normal fibroblasts and in cancer-associated fibroblasts (CAFs). During myofibroblast differentiation, fibroblasts lose CAV1 expression, which accounts for a CAF phenotype. This is characterized by lower oxidative phosphorylation (OXPHOS) and higher glycolysis. Conversely, a normal fibroblast phenotype is associated with elevated CAV1 expression, active OXPHOS and low glycolysis. Loss of CAV1 expression in fibroblasts is associated with poor outcome in breast cancer (also see text).

A pattern characterized by decreased expression or loss of CAV1, cavin 1, and CD36 in the tumor stroma of high-risk cancer patients has been emerging. However, most oncological studies focused on CAV1. Silencing of CAV1 in stroma stimulates tumor growth in a xenograft model of breast cancer [42]. Particularly, in stromal cells, the loss of CAV1 is associated with the absence of caveolae and favors a myofibroblast phenotype via transforming growth factor-β (TGFβ) signaling, oxidative stress, autophagy and glycolysis [43]. On the other hand, a low *CAV1* expression in cancer-associated fibroblasts (CAFs) is induced by oxidative stress that accounts for CAV1 degradation through autophagy, and the loss of *CAV1* expression enhances oxidative stress and autophagy in a feedforward loop [44]. Mechanistically, increasing oxidative stress also favors the transcription and stabilization of hypoxia inducing factor-1 α (HIFI-α) and nuclear factor-κB (NF-κB). In particular, the loss of CAV1 in the stroma provokes AkT activation and TGFβ1 over-expression with lower mitochondrial metabolism (oxidative phosphorylation (OXPHOS)) and higher glycolysis, and as just mentioned above, is associated with a CAF phenotype. Conversely, a high CAV1 is associated with a normal fibroblast phenotype with active mitochondrial metabolism (OXPHOS) and low glycolysis (Figure 3). In stromal cells, but not in cancer cells, a feedforward mechanism between TGFβ signaling and the loss of CAV1 expression does exist. In fact, TGFβ signaling and the loss of CAV1 expression, release matrix metalloproteinase 9 (MMP9) and increase oxidative stress. MMP9, such as loss of CAV1, promotes TGFβ activation and they both account for the generation of reactive oxygen species (ROS) in fibroblasts and increased myofibroblast motility. Increased ROS again induces TGFβ activation, CAV1 downregulation and a myofibroblasts phenotype [43].

Overall, a metabolic heterogeneity between stromal cells and cancer cells occurs. In fact, in stromal cells, altered caveolae with *CAV1* expression loss, oxidative stress and aberrant crosstalk between oxidative stress, endothelial nitric oxide (eNO), HIFI-α, and NF-κB join with increased glycolysis, while in cancer cells, increased mitochondrial OXPHOS and cell proliferation prevail. In other words, in stromal cells, CAV1 loss reduces the activity of the mitochondrial respiratory chain due to increased NO production and cholesterol accumulation in the mitochondrial membrane. This accounts for the prevailing of catabolism with glycolysis and ROS accumulation and production of intermediate metabolites, which, in a paracrine way, stimulates OXPHOS in cancer cells [43]. This metabolic heterogeneity between stromal and cancer cells is a novel elucidated mechanism governing tumor growth. Moreover, in stromal cells, monocarboxylate transporter 4 (MCT4 also known as SLC16A3) inversely correlates with loss of CAV1. MCT4 is thought to be a marker of glycolysis and lactate ejection from CAFs, and is induced by oxidative stress through activated HIFI [45]. The change of CAV1 expression between cancer cells and their normal counterparts and the effects of CAV1 expression in cancer cells on tumor aggressiveness largely differ in different cancer types. As to breast cancer, CAV1 overexpression is associated with poor clinical outcome [46]. In CAV1-knockout mouse models, tumor progression occurs and some different epithelial and stromal mechanisms seem to be involved in cyclin D1 up-regulation and increased RB phosphorylation in cancer cells and activation of the mTOR-S6 kinase pathway in myofibroblasts [47].

### 2.3. The Pathological Pathways Promoted by Hypoxia-Inducing Factors (HIFs)

HIF transcription factors family includes two main subunits, HIF-1α and HIF-2α, which are oxygen-sensitive proteins. HIF-1α is widely and differently expressed in tissues, while HIF-2α shows a much more limited expression pattern. In normal cells, under normoxic condition, HIF-α proteins are hydroxylated by prolyl hydroxylase domain (PHD) proteins [48,49]. This results in the interaction of HIF-1α with Von Hippel–Lindau (VHL) tumor suppressor gene product with the successive HIF-1α polyubiquitylation and proteasomal degradation [49,50]. In addition, Factor Inhibiting HIF (FIH) further controls transcriptional activity of HIFs escaping degradation [51]. In hypoxic conditions, as commonly occurs in cancer, the HIF-1α subunit does not interact with the VHL protein, and moves to the nucleus where it joins to the constitutively expressed HIF-β partner subunit to form a heterodimer that binds to hypoxia-responsive elements (HREs) placed in target genes promoters [52]. In cancer cells, some receptor tyrosine kinases (RTKs), such as epidermal growth factor receptor (EGFR/erbB1), HER2/erbB2/Neu, insulin-like growth factor-1 receptor (IGF1R), stem cell factor (SCF)/KIT receptor and also Notch, interleukin-6/IL-6R receptor and transforming growth factor-β/TGF-βR receptors can up-regulate the expression and/or stability of the HIF-1α subunit. These factors stimulate downstream signaling pathways including phosphatidylinositol 3 kinase (PI3K)/Akt molecular target of rapamycin (mTOR) [53] that in turn account for the overexpression and/or stability of the HIF-α subunit. A concomitant inactivation in cancer cells of tumor suppressors proteins as phosphatase tensin deleted on chromosome 10 (PTEN) and p53 can still more reduce the HIF-1α degradation and increase PI3K/Akt activation and can be responsible for HIF-α accumulation.

#### 2.3.1. HIFs as Key Regulators of Stemness

An increased expression of HIFs in cancer cells, mainly in highly tumorigenic cancer stem/progenitor cells and their differentiated progenies, may result in the transcriptional activation of genes involved in self-renewal, anaerobic glycolysis, survival, and angiogenesis. Therefore, the epithelial to mesenchymal transition (EMT) program, altered metabolism and re-expression of stem cell-like markers as Oct-3/4, Sox-2 and Nanog and the pro-angiogenic factor VEGF are induced. All these molecular transforming events account for cancer cells acquiring more malignant phenotype, neovascularization and ability to tumor metastasization [53]. Accumulation of genetic and epigenetic alterations with inactivation of tumor suppressor proteins (PTEN, p53, p27 or Rb) and activation of oncogenic signaling (RTKs, PI3K/Akt and NF-κB) trigger the malignant transformation of normal tissue and transformation of normal stem/progenitor cells to tumorigenic cancer stem/progenitor cells. However, consistent with a revised version of the cancer stem cell theory, changes in local microenvironment play a relevant role in cancer heterogeneity and development of different phenotypes from the immature cancer cells and their differentiated progenies [5,6]. Growing tumors show a disorganized vasculature and hypoxic intra-tumoral regions, which need adaptation to cancer cells for their survival [54,55]. This induces changes to more aggressive phenotypes and survival advantages. At the level of cancer stem cells and their differentiated progenies, hypoxia and increased HIF-1α expression and activity promote up-regulation of different stemness elements and survival signaling factors [53]. Particularly, pluripotency promoting factors (Oct-3/4, Sox-2 and Nanog), EMT program (EGFR, CXCR4, snail and twist) glucose uptake, glycolytic enzymes, microRNAs (miRNAs) and drug resistance promoting elements are induced. These signaling factors take part and sustain central functions, such as self-renewal ability, energy supply by increased aerobic, anaerobic glycolysis, the spread and involvement of secondary sites, and the resistance to therapy [53]. All of this is supported by the findings of co-expression of HIF-1α and the CD44^+^/CD24^−^/low phenotype (self-renewal ability) associated with worse prognosis of breast cancer patients [56]. Moreover, the over-expression of Jagged2 and nuclear Notch intracellular domain (EMT program induction) in hypoxic regions occurs at the invasive front of breast cancer tissues.

#### 2.3.2. HIFs as Regulators of Cancer Progression and Metastasization

As to the metastasization capability, it has been found that CD44^+^/CD24^−^/low BCSCs over-expressing HIF-1α and mesenchymal markers N-cadherin and vimentin had a higher clonogenic and mammosphere-forming abilities than their differentiated progenies [57,58]. These abilities were reported to be induced by activated CAFs that released SDF-1, which, in turn, stimulated CD44^+^/CD24^−^/low BCSCs expressing the cognate receptor CXCR4 and neovascularization [59]. Moreover, hypoxic cancer cells at the level of primary and secondary breast tumors seem to play a relevant role in the formation of pre-metastatic niches and secondary lesions in the hypoxic bone microenvironment [53]. In fact, HIF-1α can overexpress and secrete lysyl oxidase (LOX), lysyl oxidase-like 2 (LOXL2) and LOXL4 in hypoxic breast cancer cells [60,61]. They induce the formation of pre-metastatic niches by extracellular matrix (ECM) remodeling and promote the recruitment of CD11b+ bone marrow-derived cells (BMDCs) [60,61]. In addition, the CXCR4 over-expression in breast cancer cells is thought to be important in driving the metastatic involvement towards organs as bone and lungs that secrete many ligand molecules acting as a chemo-attractant gradient [62]. Within the hypoxic bone microenvironment, interactions between stromal cells and breast cancer cells are likely to govern their dormancy, self-renewal capability, the formation of osteolytic metastases, and the resistance to treatment. Specifically, this may occur through the release by both types of cells of growth factors and cytokines, such as SDF-1, TGF-β1 and BMPs, and over-expression of HIF-1α, NF-κB, vascular cell adhesion molecule-1 (VCAM-1) and Notch in breast cancer cells [53]. It has been reported that BCSCs show a higher rate of bone metastases, compared with parental breast cancer cell line, expressed higher levels of CD44, CXCR4, and osteopontin markers [63]. Additionally, it has been found that EMT program promotion, by the increased HIF-1α and TGF-β signaling, induced over-expression of CXCR4 and VEGF in breast cancer cells collaborated in their diffusion and secondary bone involvement [64].

#### 2.3.3. Molecular Signaling Mechanisms and Novel Interconnections Involving HIF-1 Pathway

Some recent experimental studies have elucidated novel interconnections or molecular signaling mechanisms involving HIF-1α pathway. In one of them [65], the oxygen sensor hypoxia-inducible factor prolyl hydroxylase 2 (PHD2), which is thought to be the principal HIF-1α regulator, has been found to correlate with EGFR in samples from breast cancer patients positively, thus showing a direct crosstalk between PHD2 and EGFR-mediated tumorigenesis in breast cancer for the first time. In another [66], a new signaling pathway involving an activator of HIF-1α, the Munc18-1-interacting protein 3 (Mint3), has been described. This protein, which works in cancer cell macrophages and fibroblasts, even during normoxia, regulated the relationship between cancer cells and stromal cells. Particularly, it has been found that Mint3 and HIF-1α-induced in fibroblasts L1 cell adhesion molecule (L1CAM). The L1CAM Mint3-mediated expression in fibroblasts on turn stimulated in cancer cells the ERK signaling molecular pathway through integrin α_5_β_1_, thus promoting cancer cell proliferation and tumor growth. In another experimental study [67], fructose-1,6-biphosphatase 1 (FBP1) was down-regulated in MDA-MB-468 basal-like cell line, and its over-expression under hypoxic condition was significantly associated with decreased tumor growth and migration as well as glycose consumption and lactate production likely through HIF-1α inhibition and reduced mRNA levels of pyruvate dehydrogenase kinase 1 (PDK1), lactate dehydrogenase A (LDHA), glucose transporter 1 (GLUT1), and vascular endothelial growth factor (VEGF). In a further one [68], in human breast cancer cells, cancer-associated oxidoreductase ERO1-α over-expression increased HIF-1α protein expression. They both resulted in inducing PD-L1 overexpression at mRNA and protein level thus uncovering a novel mechanism of immune escape. Another study [69] analyzed the effects of hypoxia on ER-α protein, mRNA and transcriptional activity in a panel of ER α-positive breast cancer cell lines. Stabilized HIF-1α-induced loss of ER-α protein in all cell lines through proteolysis rather than transcriptional repression. Thus, the authors hypothesized that inhibitors of HIF-1α or proteasome could stabilize ER-α expression in vivo and synergize with endocrine therapies to overcome or delay endocrine resistance. In two very recently published experimental studies, the relevant role of HIF-1α in triple negative breast cancer (TNBC) was highlighted. In the former conducted in human TNBC MDA-MB-231 and Hs578T and non-TNBC MCF-7 and BT474 tumor-bearing mice, anti-angiogenic treatment promoted cancer invasion via vasculogenic mimicry in the microcirculation of malignant tumors.HIF-1α, MMP2, VE-cadherin, and twist1 were expressed at a higher level in human TNBC compared with non-TNBC. Moreover, the clinical significance of this upregulation was validated in 174 human breast cancers [70]. The latter reminded that “high HIF-1α expression is associated with aggressiveness of the cancer. However how HIF-1α is regulated and how HIF-1α induces aggressive phenotype are not completely understood in TNBC”. Thereafter, it reported on the capability of low-dose farnesyltransferase inhibitor (FTI) to suppress HIF-1α and snail expression in TNBC MDA-MB-231 cells in vitro. The authors concluded that FTIs and snail could improve the aggressive phenotype of TNBC by inhibiting the HIF-1α-snail pathway [71].

#### 2.3.4. HIFs-NF-κB Crosstalk and Inflammation in Cancer

**Figure 4:**
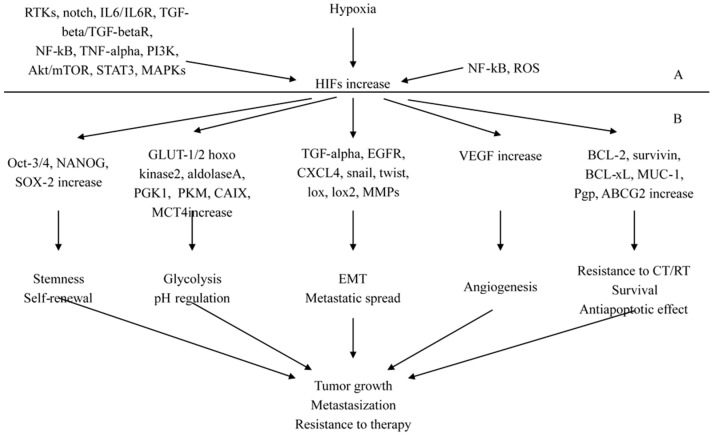
Hypoxia inducible factors (HIFs): (**A**) signaling mediated involvement in the regulation and activation of HIFs; and (**B**) signaling factors and programs promoted by HIFs. BCRP/ABCG2: breast cancer resistance protein; CAIX: carbonic anhydrase; EGFR: epidermal growth factor receptor; GLUT: glucose transporter; IL-6: interleukin-6; MAPK: mitogen-activated protein kinase; MCT-4: monocarboxylate transporter-4; MIC-1: macrophage inhibitory cytokine-1; MMPs: metalloproteinases; mTOR: molecular target of rapamycin; NF-κB: nuclear factor κB; RTK: receptor tyrosine kinase; PI3K: phosphatidyl inositol-3 kinase; PGK1: phosphoglycerate kinase-1; PKM: pyruvate kinase M; Pgp: P-glycoprotein; ROS: reactive oxygen species; TGF-β: transforming growth factor-β; TNF-α: tumor necrosis factor-α; STAT3: signal transducer activator of transcription-3; VEGF: vascular endothelial growth factor; EMT: epithelial to mesenchymal transition; CT: chemotherapy; RT: radiotherapy; LOX: lysyl oxidase; MUC-1: mucin-1; CXCL4: chemokine ligand 4; BCL-2: B-cell lymphoma-2 (also see text).

Nuclear Factor binding to the enhancer element of the immunoglobulin κ light chain of activated B cells (NF-κB) is the collective name of a family of transcription factors [72], and NF-κB pathway is mainly recognized to be activated in inflammation [7]. A bi-directional crosstalk at different levels [73] does exist between HIF-1α and NF-κB so that NF-κB induces HIF-1α, while HIF-1α governs NF-κB pathway. Inflammation in tumor is associated with a close collaboration between HIF-1α and NF-κB. Particularly, HIF-1α contributes to an inflammatory response through induction of some pro-inflammatory chemokines and cytokines [74] and inflammation promote an increased NF-κB activity [74]. Moreover, *IL6*, *MMP9*, cyclooxygenase 2 (*COX2*) and *Bcl-2* are some among others genes involved in tumorigenesis that is the target of both of HIF-1α and NF-κB [75]. In hypoxic conditions, PHDs and FIH are 2-OG-dependent dioxygenases enzymes, which work as oxygen sensors that stabilize HIF-1α. In the same conditions, these enzymes and other dioxygenases can confer oxygen sensitivity to further molecular pathways including NF-κB. This mechanism is supported by findings showing new potential FIH and PHD targets. Particularly, OTU dDe-ubiquitinase, Ubiquitin, Aldehyde Binding 1 (OUTB1), which is an upstream regulator of the NF-κB pathway, has been found to be hydroxylated by FIH [76] and is thought to provide links to NF-κB and oxygen sensing [76]. Moreover, other enzymes are likely required for hypoxia-induced NF-κB activity [77]. Similarly to FIH, there is the evidence that in a hypoxic condition, PHD governs NF-κB induction, although a direct oxygen sensing mechanism has not yet elucidated. Moreover, in different cell types PHDs have been reported to contrast NF-κB activity [51]. Overall, these investigations suggest that NF-κB regulation by PHDs is cell type and context dependent. Jumonji C (JmjC) domain-containing proteins, many of which are 2-OG dioxygenases are enzymes working as protein demethylases and are often deregulated in many cancers [78]. A few investigations have reported on high levels of histone methylation marks during prolonged hypoxia attributed to impair the activity of these enzymes [79,80]. Many of these enzymes are HIF-1α targets and hypoxia-inducible, thus suggesting the existence of a negative feedback mechanism helpful in a JmjC histone demethylase compromised environment to allow cells to reset their oxygen sensing and response, and to restore normoxia after prolonged hypoxia. However, they also represent a feedback loop of NF-κB that governs its own activity, and it is likely that in the hypoxic condition they provide another mechanism of cross-talk between HIF-1α and NF-κB activity. In fact, non-histone targets for these enzymes have been found, and in particular, it has been discovered that the expression of some target genes of p65 (ReIA), which takes part of NF-κB pathway is inhibited through demethylation by the KDM2A JmjCs enzyme [81,82]. Interestingly, it has been reported that some methylation sites in this p65 are oppositely regulated by nuclear receptor binding SET domain protein 1 (NSD1) and KDM2A and that KDM2A is NF-κB [82] and hypoxia-inducible [83]. In the hypoxic microenvironment, inactivation of the NF-κB inhibitor IkB α through TAK1 (transforming growth factor-β-activated kinase 1)-IKK mediated phosphorylation has been reported [77,84] and can promote NF-κB activation. However, in hypoxic condition ubiquitination is replaced with sumoylation. Thus, because sumoylation is relevant for hypoxia-induced NF-κB activation, it is currently thought that it should be intensively investigated [51]. Elements and signaling pathways involved in the stabilization, regulation, and activation of hypoxia-inducible factors are schematically showed in Figure 4.

### 2.4. Disturbances in the Apoptotic Machinery

A few studies have linked breast cancer progression and drug resistance to over-expression of pro-survival factors including Bcl-2, Mcl-1 and other BH3 family members [85]. The BCL-2, MCL-1, and BCL-XL pro-survival proteins and the pro-apoptotic BH3-only ligand BIM are contemporaneously over-expressed in a significant proportion of the different breast cancer subtypes including the more aggressive basal-like [86]. In spite of the evidence of a multifactorial role in cancer biology by mechanisms other than apoptosis [87,88,89,90], a principal BCL-2 protein function is to heterodimerize with pro-apoptotic members of the BH3 family to inhibit mitochondrial pore formation and cytochrome c release that commonly triggers apoptosis. A study [86] explored the role of BCL-2 as a potential therapeutic target in breast cancer and showed a sensitization of BCL-2 expressing breast cancers to chemotherapy by the BH3 mimetic ABT-737. The BH3 mimetics are the most potent small molecule inhibitors of the BCL-2 subfamily, and ABT-737 binds with high affinity to BCL-2, Bcl-xl, and Bcl-w, although it does not bind to Mcl-1 [91]. A panel of primary breast tumor xenografts was generated in immune-compromised mice, and recipients received either ABT-737, docetaxel or a combination. While treatment with ABT-737 alone was ineffective, the combination was associated with increased apoptosis and dissociation of BIM from BCL-2, suggesting that ABT-737 sensitized the tumor cells to docetaxel. The eukaryotic 26S proteasome is a large complex responsible for identification and docking of polyubiquitinylated proteins. It includes three catalytic β-subunits reacting with peptide bonds of substrates. It governs the turnover of cyclins and cyclin-dependent kinase inhibitors; in addition, the ubiquitin-proteasomal complex regulates the turnover of transcriptional factors as NF-κB, p53, BCL-2 family members and others. Tumor tissue has an increased proteasome activity that induces degradation of tumor suppressor proteins. This results in cancer cell survival and proliferation as well as the arising of resistance to apoptosis. Conversely, the proteasome inhibition accumulates Bax (but not BCL-2) in mitochondria, which results in an increased ratio of Bax/BCL-2 with the following cytochrome c release and apoptosis induction [91]. A pilot study included fourteen patients with loco-regional recurrence following breast-conserving therapy for early breast cancer. An altered proteasome signaling was supposed to be linked with radio-resistance [92]. These patients were compared with fourteen patients who were disease-free at 10 years. A decreased expression of the 26S proteasome was significantly associated with radioresistance suggesting that the 26S proteasome may be associated with response to radiotherapy.

Alterations of Signaling Pathways in Some Specific Biological Mechanisms Involved in Breast Cancer Progression (Table 1)

The heparin/heparin-sulfate interactome, over-expression of miRNA-378a-5p, restriction of luminal, basal epithelial cells, and fatty-acid synthesis.

In one study [93], it has been found that perturbation of the heparin/heparin-sulfate interactome of human breast cancer cells regulates pro-tumorigenic effects in association with PI3K/Akt and MAPK/ERK signaling. Namely, heparan sulfate-proteoglycans (HSPGs) through their polyanionic heparan sulfate (HS) interact with proteins placed on the cell surface or in the extracellular matrix membrane to form a functional network called heparin/HS interactome. Authors conclude that the HS associated activity promotes a tumorigenic phenotype and cell adhesive, invasive and migratory properties. In addition, they found that following heparin treatment, the innate TGF-β activity of MCF-7 cells decreased through specific inhibition of the TGF-β-smad signaling pathway. In another experimental study [94], over-expression of miRNA-378a-5p correlated with breast cancer tumorigenesis in vivo. A premature inactivation of the spindle assembly checkpoint (SAC), which is an evolutionarily conserved safeguard mechanism controlling the fidelity of mitosis, likely accounted for the induced numerical chromosome changes in cells. Additionally, miRNA-378a-5p over-expression induced receptor tyrosine kinase-MAP-Kinase pathway signaling, which occurred concomitantly with suppression of Aurora B kinase that is a critical regulator of SAC. Moreover, in the breast cancer cells in vivo miRNA-378a-5p over-expression was associated with the most aggressive and poorly differentiated molecular subtypes. Two other studies focused on the capability to overcome the lineage restriction of luminal and basal epithelial cells that are the main cells of origin of breast cancer. In postnatal mammary gland in mice, almost ever one lineage switches to the other lineage. The former investigation from Koren et al. [95] used inducible Cre recombinase transgenes driven by the Lgr or K5 promoters expressed by basal epithelial cells, and the latter by Van Keymeulen et al. [114] used K8 promoter expressed by luminal epithelial cells to drive expression of fluorescent fate-tracking reporter alleles. Lacking any manipulation luminal cells did not transform in basal cells and vice versa, while following the introduction of the activating mutant p110α ^H1047R^ in luminal or in basal epithelial cells, transformation in basal or luminal epithelial cells respectively occurred. These findings highlight the existence of a relationship between genetic mutation hyper-activating the PI3K pathway and promotion of stemness and tumor heterogeneity. In highly proliferating cancer cells, unlike in normal cells, de novo fatty acid synthesis is increased due to the need of fatty acids for membrane and energy production. Acetyl-CoA carboxylase (ACACA) and fatty-acid synthase (FASN) are the two primary enzymes involved in de novo fatty-acid synthesis, while 3-hydroxy-3 methylglutaryl-CoAreductase (HMGCR) and CYP27B1 are the key enzymes for de novo cholesterol synthesis and calcium homeostasis respectively. An experimental study [96] showing gene expression profile in presence/absence of has-miR-195 in breast cancer cells found that ACACA, FASN, HMGCR, and CYP27B1 were the direct targets of has-miR-195 and Ingenuity Pathway Analysis (IPA) evidenced mitochondrial dysfunction with involvement of fatty acid metabolism and xenobiotic metabolism signaling. Moreover, ectopic expression of has-miR-195 in MCF-7 and MDA-MB-231 cells not only was found to be significantly associated with decreased cholesterol and triglyceride levels but also with diminished proliferation, invasion, and migration. The authors conclude that has-miR-195 and miR-195 target genes can provide new options for breast cancer treatment.

### 2.5. Recently Unraveled Molecular Pathways Related to Epithelial to Mesenchymal Transition (EMT)

In the report mentioned immediately above, has-miR-195 also decreased the expression of the mesenchymal markers and increased that of epithelial ones. Three other experimental studies described novel mechanisms involved EMT or vice versa. In one of them [97], silencing of estrogen receptor in MCF-7 breast cancer cells by siRNA gave rise to estrogen/tamoxifen-resistant cells defined as PII. Changes were found in about 2500 unique sequences with about 1270 of them up-regulated as well as increased motility, a switch from keratin/actin to vimentin based cytoskeleton and capability to diffuse to simulated components of the ECM. The authors hypothesize that induced loss of estrogen receptor in estrogen/anti-estrogen sensitive cells provokes the loss of endocrine dependence and switches cells from an epithelial to a mesenchymal phenotype. In cancers including breast cancer, although the mechanism remains unknown, altered monoamine oxidase-A (MAO-A) expression and depression correlate with poor outcome. Moreover, while commonly MAO-A mRNA is inhibited, MAO-A protein and serotonin concentrations are increased. In an experimental study [98], the action of the monoamine oxidase-A (MAO-A) inhibitor clorgyline was evaluated in the epithelial ER-positive MCF-7 and the post-EMT (mesenchymal) ER-negative MDA-MB-231 human breast cancer cell lines. Any effect of clorgyline on metastatic behavior depended on the cell’s EMT condition rather than the ER status, and clorgyline promoted EMT in MDA-MB-231 breast cancer cell line through a non-canonical mechanism. In a further study [115], the histone H2A isoform Hist2h2ac has been reported to be a novel regulator of proliferation and EMT in mammary epithelial and in breast cancer cells. In the study, the canonical Hist2h2ac was found only in undifferentiated/proliferating cells; Hist2h2ac mRNA was induced by MEK1/2 or PI3-K activation in HC11 and EpH4 mammary epithelial cells and in MC4-L2 and T47-D breast cancer cells. Hist2h2ac silencing inhibited EGF-induced Zeb-1 expression and E-cadherin down-regulation, and they were both reverted by Hist2h2ac re-expression. The authors claim that this is the first time histone isoform Hist2h2ac has been identified downstream of the EGFR pathway where it takes part to deregulate target genes.

### 2.6. HER-2/Neu Gene Amplification and Protein Expression and the Expression of Other Members of the Epithelial Growth Factor Receptor Family

The crosstalk of ER α with HER2 and the other members of the epithelial growth factor receptor (EGFR) family are well known, widely reported, and are commonly considered among the reasons responsible for breast cancer progression following the development of endocrine resistance [99,100,101]. Moreover, a recent review has reported on breast cancer progression due to the mechanisms of resistance to anti-HER2 therapies in HER2+ breast cancer [102], and here the main data are briefly summarized. The PI3K/Akt/mTOR pathway is a well-recognized effector of HER2 signaling. Trastuzumab is an anti-HER2 monoclonal antibody while lapatinib is a tyrosine kinase inhibitor that reversibly inhibits phosphorylation of EGFR, HER2, Erk-1 and-2 and AKT kinases. There are findings that point out that either PIK3CA mutation or PTEN loss, in spite of HER2 blockage, foster a treatment escape mechanism through the PI3K/Akt/mTOR pathway. Therefore, trials aiming to target the PI3K/Akt/mTOR pathway at different level together with HER2 are ongoing. In these trials, specific inhibitors of different isoforms of PI3K, pan PI3K inhibitors, Akt-inhibitors or mTOR-inhibitors are evaluated in different settings commonly in association with trastuzumab and chemotherapy to delay breast cancer progression and/or overcome resistance to trastuzumab. A truncated fragment of the HER2 receptor called p95HER2 and insulin-like growth factor-1 receptor (IGF-IR) have been recognized as other biomarkers of resistance to anti-HER2 therapy. In addition, a crosstalk between HER2 and IGF-IR has been documented in trastuzumab-resistant cells, and the inhibition of IGF-IR tyrosine kinase activity can account for diminished HER2 phosphorylation and restoration of trastuzumab sensitivity [103,104]. cMet over-expression or Met aberrations, Src activation, and the level of the immune response are described as three further potential mechanisms promoting breast cancer progression during anti-HER2 therapy [105,106,107]. cMET is a receptor tyrosine kinase that, upon binding with the hepatocyte growth factor (HGF), promotes cell proliferation through MAPK, PI3K, and signal transducer and activator of transcription (STAT). Src is a proto-oncogene encoding for the non-receptor protein kinase Src that extensively interacts with transmembrane receptor tyrosine kinases (RTKs) as HER1 and HER2. Src activation accounts for tumor progression following both acquired and de novo resistance to trastuzumab. Moreover, resistance to lapatinib has been found in Src activated cell lines. Regarding the relationship between immune response and sensitivity to anti-HER2 therapies, experimental data and findings from the GeparQuattro trial are mentioned. Notably, in the GeparQuattro trial, a direct positive correlation was observed between the levels of tumor-infiltrating lymphocytes and the number of patients with pCR. Impaired HER2 accessibility due to mucin 4 (MUC4) [108], HER2 reactivation through the acquisition of the HER2 L755S mutation [109] and activation of STAT3/HIF-1α/Hes-1 axis [110] are other mechanisms elucidated in three more different recent experimental investigations. All these mechanisms were able to promote tumor progression and resistance to trastuzumab or to HER2-targeted therapy in HER2 over-expressing cancer cells. Moreover, in another review article [111], de-escalation approach, that combines anti-HER2 treatment with only a single chemotherapy or in the absence of any chemotherapy, is suggested as a promising therapeutic strategy to be explored in HER2 amplified tumors. In one phase I of two clinical trials, significant decrease in the p-Src expression on epidermal keratinocytes on sequential skin biopsies occurred using the Src kinase inhibitor dasatinib with trastuzumab, and paclitaxel as first-line therapy for patients with HER2 over-expressing advanced breast cancer [112]. In the other phase I/II study [113], patients with advanced HER2-amplified breast cancer received trastuzumab and suberoylanilide hydroxamic acid (SAHA; vorinostat), a small molecule that is known to inhibit histone deacetylase and weaken signaling pathways which induce tumor progression and trastuzumab resistance. However, in these patients, there was no evidence that SAHA addiction reverses trastuzumab resistance.

## 3. Conclusions

Breast cancer is a “work in progress” being the object of many experimental and clinical investigations. Here, we focus on recently elucidated signaling pathways related to some general principles and some other more specific biological mechanisms involved in breast cancer progression. These advances in basic research are necessary to sustain the efforts based on the new genotyping techniques for more accurate prognostic and predictive biomarkers. As to this, the tumor and circulating DNA assay in liquid biopsies is the new frontier for an easier and harmless personalized management of breast cancer disease.

## Figures and Tables

**Table 1:** Some more specific biological mechanisms and signaling pathways involved in breast cancer progression.

Biological Component(s)	Mechanism	Outcome	References
Heparin/heparin sulfate interactome	Increased PI3K/Akt, MAPK/ERK signaling and TGF-β activity	Tumorigenic phenotype, cell adhesive, invasive properties	[93]
miR-37pa-5p overexpression	Inactivation of SAC through induced receptor tyrosine kinase-MAPK pathway and suppression of Aurora kinase	More aggressive and poorly differentiated molecular subtypes	[94]
Lineage restriction	Genetic mutation of hyperactivating to PI3K pathway in luminal or basal epithelial cells	Induction of stemness and tumor heterogeneity	[95]
has-miR-195 and miR-195	Decreased cholesterol and triglycerides with mythocondrial dysfunction and involvement of xenobiotic metabolism signaling	Decreased proliferation, invasion and migration	[96]
ER-α	ER-α loss	EMT induction	[97,98]
MAO-A	MAO inhibitor (clorgyline) through non-canonical pathway		
Hist2h2ac histone isoform	MEK1/2 or PI3K activation		
ER α HER2 amplification and expression of other members of EGFR family	ER-α/HER2 cross-talk, PI3K/Akt/mTOR pathway escape (PI3KCA mutation/PTEN loss, HER2/IGF1R cross-talk), c-met overexpression, src activation, low TILs level	Breast cancer progression	[99,100,101,102,103,104,105,106,107,108,109,110,111,112,113]

SAC: spindle assembly checkpoint; ER: estrogen receptor; MAO-A: monoamine-oxydase-A; EMT: epithelial to mesenchymal transition; EGFR: epidermal growth factor receptor; TILs: tumor-infiltrating lymphocytes.
